# Inhibition of Porcine Epidemic Diarrhea Virus by Cinchonine *via* Inducing Cellular Autophagy

**DOI:** 10.3389/fcimb.2022.856711

**Published:** 2022-06-14

**Authors:** Jingping Ren, Wei Zeng, Changsheng Jiang, Chang Li, Chengjun Zhang, Hua Cao, Wentao Li, Qigai He

**Affiliations:** ^1^ State Key Laboratory of Agricultural Microbiology, College of Veterinary Medicine, Huazhong Agriculture University, Wuhan, China; ^2^ The Cooperative Innovation Center for Sustainable Pig Production, Huazhong Agricultural University, Wuhan, China

**Keywords:** porcine epidemic diarrhea virus, cinchonine, antiviral, autophagy, endoplasmic reticulum (ER) stress

## Abstract

Porcine epidemic diarrhea virus (PEDV) could cause lethal diarrhea and dehydration in suckling piglets, which can adversely affect the development of the global swine industry. The lack of effective therapeutical and prophylactic treatment especially for PEDV variant strains underlines the importance of effective antiviral strategies, such as identification of novel antiviral agents. In the present study, the antiviral activity of cinchonine against PEDV was investigated in Vero CCL81 and LLC-PK1 cells at a non-cytotoxic concentration determined by Cell Counting Kit-8 assay *in vitro*. We found that cinchonine exhibited a significant suppression effect against PEDV infection and its inhibitory action was primarily focused on the early stage of PEDV replication. Moreover, we also observed that cinchonine could significantly induce autophagy by detecting the conversion of LC3-I to LC3-II by using western blot analysis. Cinchonine treatment could inhibit PEDV replication in a dose-dependent manner in Vero CCL81 cells, while this phenomenon disappeared when autophagy was attenuated by pre-treatment with autophagy inhibitor 3MA. Consequently, this study indicated that cinchonine can inhibit PEDV replication *via* inducing cellular autophagy and thus from the basis for successful antiviral strategies which potentially suggest the possibility of exploiting cinchonine as a novel antiviral agent.

## Introduction

Porcine epidemic diarrhea (PED) was first reported in the United Kingdom in 1971 caused by porcine epidemic diarrhea virus (PEDV) (Wood, 1977), and is one of the most important digestive tract infectious diseases of pigs. PEDV is an enveloped virus containing a single-stranded positive-sense RNA genome (Wang et al., 2020). It belongs to the genus *Alphacoronavirus* in the *Coronaviridae* family, and includes 5’UTR-ORF1a/1b-S-ORF3-E-M-N-3’UTR, encoding 16 different non-structural proteins and 4 structural proteins (Kocherhans et al., 2001; Song and Park, 2012). PEDV infection is often characterized by severe enteritis that results in acute diarrhea followed by vomiting and dehydration (Pensaert and Martelli, 2016), causing high morbidity and mortality in neonatal piglets under 0-7-day-old. Over the past ten years, PED has significantly threatened the development of the global pig industry, leading to substantial economic losses and public health security hazard (Huang et al., 2013).

To protect against PEDV infection, the livestock industry has made substantial efforts to control PEDV as the genetic variation emerged especially in the *S* gene (Jung and Saif, 2015). Immunization prevention is one of the most effective means that can effectively control viral infections. Inactivated vaccines and attenuated vaccines based on PEDV CV777 strain are currently diffusely used to prevent PEDV infection. However, traditional vaccines fail to provide competent protection to pigs against the PEDV variant strains, thus making it important to develop novel antiviral agents against PEDV infection for further prophylaxis and treatment (Li et al., 2020). A number of studies have found various natural compounds and ingredients that can exhibit significant antiviral activities against PEDV, including quercetin (Choi et al., 2009; Song et al., 2011; Li et al., 2020), aloe (Xu et al., 2020), griffthsin (Li et al., 2019) and 2-Deoxy-D-glucose (Wang et al., 2014). Although there are many reports about the existence of different anti-PEDV drugs, they have not been commercially used in the clinic until now. Cinchonine is a natural compound extracted from Cinchona and *Ramijia* (Rubiaceae) bark (Rankovic et al., 2019), and can exhibit synergistic effects with quinine, quinidine, and cinchonidine and among which cinchonine shows highest activity and low toxicity (Nobili et al., 2006). It has been recognized that cinchonine possesses antimalarial (Sowunmi et al., 1990), antiobesity (Shah et al., 1997; Shah et al., 1998; Jung et al., 2012), antiplatelet (Shah et al., 1998) and antimultidrug resistance but its performance as an antiviral agent has not been reported. Therefore, this paper attempts to investigate whether cinchonine can display antiviral activities and explore its underlying mechanisms of action.

Autophagy is a lysosome-dependent degradation process, which is primarily characterized by the formation of autophagosomes, and the long-lived proteins and damaged organelles are transported to lysosomes for degradation and circulation (Sun et al., 2014; Wang et al., 2019). Nutrient deficiency, high temperature, oxidative stress, endoplasmic reticulum(ER) stress, viral infections, or pharmacological agents could effectively induce autophagy to sustain the cellular homeostasis (Mizushima et al., 2010). It has been established that microtubule-associated protein 1 light chain3 (LC3) is the marker of autophagosomes, and is the most commonly detected autophagy-related protein (Suzuki et al., 2001; Klionsky et al., 2003). Generally, the conversion of LC3-I to LC3-II could be monitored by Western Blot to reflect the levels of autophagy (Mizushima et al., 2010). Increasing studies have indicated that complex connection relationship exists between autophagy and viral infections. Autophagy acts as an antiviral mechanism that can inhibit infection by packaging viruses in the autophagosome (Liang et al., 1998; Liu et al., 2005), whereas many pathogens utilize autophagy for their replication (Dreux and Chisari, 2010). Some viruses, such as herpes simplex virus type 1 (HSV-1) (Orvedahl et al., 2007; Liu et al., 2021) and human cytomegalovirus (HCMV) (Chaumorcel et al., 2008) have evolved different strategies to disrupt the process of autophagy for their survival. However, viruses can also functionally utilize autophagy to ensure their own infection, especially for RNA viruses, such as porcine reproductive and respiratory syndrome virus (PRRSV) (Sun et al., 2012), coxsackievirus B3 (Wong et al., 2008), and Newcastle disease virus (NDV) (Sun et al., 2014). As for PEDV, evidence suggested that PEDV can interact with the cellular autophagy pathway to enhance its replication in Vero cells (Guo et al., 2017; Zou et al., 2019). However, rapamycin was shown to restrict PEDV infection *via* inducing autophagy in porcine jejunum intestinal epithelial cells (IPEC-J2) (Ko et al., 2017).

In this study, we have observed that autophagy-induced by cinchonine is actively involved in inhibiting PEDV replication *in vitro*. Our results also suggested that cinchonine may exhibit additional biological properties besides what was previously reported, and it might provide a novel anti-PEDV drug which can be beneficial for the swine industry.

## Materials and Methods

### Cells, Virus and Antibodies

The PEDV YN15 (GenBank accession No. KT021228.1), GD (GenBank accession No. KU985230), CV777 (GenBank accession No. AF353511.1) and DR13 (GenBank accession No. JQ023161) strains were preserved in our laboratory. Vero CCL81 (African green monkey kidney) cells, ST (Swine testis) cells and LLC-PK1 (Porcine kidney) cells were cultured and maintained in Dulbecco’s modified Eagle’s medium (DMEM, Gibco) containing 8% fetal bovine serum (FBS, Suero, Industria Argentina) and incubated at 37°CC with 5% CO_2_. Reed-Muench method was used to calculate the 50% tissue culture infectious doses (TCID_50_) to determine the virus titer. Cinchonine, 3-Methyladenine (3MA), Tunicamycin and 4-Phenylbutyric acid (4-PBA) were purchased from MedChemExpress (MCE) and dissolved in DMSO for preservation. The β-actin polyclonal antibody, LC3 polyclonal antibody and HRP-conjugated AffiniPure Goat Anti-Rabbit IgG (H+L) were purchased from ABclonal Technology (China, Wuhan). The Alexa 488-labeled anti-mouse antibody was purchased from Antgene. Monoclonal antibody (MAb) against the PEDV N protein and MAb against the PEDV S were developed in our laboratory.

### Cytotoxicity Assay

The cytotoxicity of cinchonine was evaluated in Vero CCL81 cells, ST cells and LLC-PK1 cells by using Cell Counting Kit-8 (CCK-8, Beyotime Biotechnology, China). The cells seeded in 96-well cell culture plate were treated with increasing concentrations of the cinchonine ranging from 0 to 200 μM. The treated cells were then incubated for 48 h at 37°C in 5% CO_2_. After incubation, the medium was removed, and the cells were washed with PBS three times. Thereafter, 110 μL CCK8 solution (10 μL CCK8 regent in 100 μL DMEM) was added to the wells and then incubated for 2 h at 37°C with 5% CO_2_ in the dark. The optical density (OD) value was measured at the wavelength of 450 nm with a microplate reader (Victor NIVO 3S, USA). The percentage of cell viability was calculated by using the following formula: Cell viability= [As – Ab]/[Ac – Ab]. As: the OD_450_ value of the cinchonine treated wells (wells with cinchonine treated cells and CCK8 solution); Ab: the OD_450_ value of the mock wells (wells with CCK8 solution and DMEM); Ac: the OD_450_ value of the wells without cinchonine (wells with cells and CCK8 solution only).

### RNA Extraction and Quantitative Real-Time PCR

Total RNA was extracted from Vero CCL81 cells by using a Simply P Total RNA Extraction Kit (Bioflux, China) and reverse transcribed into cDNA using HiScript^®^ II Q RT SuperMix (Vazyme, China), following the manufacturer’s instructions. The quantitative real-time PCR assay for quantifying the PEDV genome was carried out by using the following primer and probe sequences: PEDV forward primer: 5’- CGTACAGGTAAGTCAATTAC-3’; PEDV reverse primer: 5’-GATGAAGCATTGACTGAA-3’; and PEDV Taq-Man^®^ probe: FAM-TTCGTCACAGTCGCCAAGG-TAMRA. Quantitative real-time PCR was performed using Hieff UNICON^®^ qPCR TaqMan Probe Master Mix (YENSEN, China). Each reaction was performed in triplicate.

### Western Blot Assay

The cells grown on the 12-well plate were lysed with 100 μL RIPA lysis buffer (Beyotime Biotechnology, China) on ice for 30 min. After adding the loading buffer, the cell lysates were boiled for 10 min and then centrifuged at 12,000 r/min for 5 min at 4°C. The protein extract was loaded on SDS-PAGE and then transferred onto a polyvinylidene fluoride (PVDF) membranes. The membranes were blocked with 5% skimmed milk for 2 h at room temperature. After blocking, the membranes were incubated with primary antibodies overnight at 4°C, followed by being washed three times with TBST. Then the membranes were incubated with HRP-linked secondary antibody for 2 h at room temperature. The target protein was exposed with an Omni-ECL™ Pico Light Chemiluminescence Kit (EpiZyme, China).

### TCID_50_ Assay

Vero CCL81 cells in 96-well culture plate were inoculated with serial 10-fold dilutions of virus. After inoculation, the plates were incubated at 37°C with 5% CO_2_ until complete cytopathic effect (CPE) was completed. For the YN15 strain, the wells with syncytium formation, and the specific cytopathic effect caused by YN15, were classified as PEDV-positive. As for the DR13 strain, IFA staining was performed and those with specific staining were considered to be PEDV-positive. Eventually, the PEDV titration was calculated by using 50% tissue culture infectious doses (TCID_50_) following the Reed–Muench method (Wang et al., 2020).

### Indirect Immunofluorescence Assay (IFA)

Vero CCL81 cells in 48-well culture plate were washed with PBS for three times and then fixed with 4% paraformaldehyde for 15 min at room temperature. Vero CCL81 cells were then permeated with ice cold methanol, and blocked in PBS containing 5% bovine serum albumin (BSA) for 30 min at 37°C. After blocking, the cells were incubated with anti-PEDV S protein antibodies diluted in PBS for 1 h at 37°C. Thereafter, the cells were incubated with Alexa 488-labeled anti-mouse antibody for 30 min at 37°C. After washing with PBS, the cells were visualized using a Nikon inverted fluorescence microscope.

### Time-Of-Addition Assay

Vero CCL81 cells in 24-well culture plate were washed with PBS for three times and infected with the PEDV at a MOI of 0.01. Then, 100 μM cinchonine was added into infected cells at the different time periods after PEDV infection: 0-10, 0-2, 2-4, 4-6, 6-8 and 8-10 h, and the cultures were thereafter incubated for 24 h at 37°C.

### Dose-Of-Addition Assay

Vero CCL81 cells were seeded in 24-well plate, cultured for 48 h, and then infected with PEDV (MOI of 0.01). Serially diluted cinchonine was then added to the culture medium of the cells (final concentrations used were 20, 40, 60, 80 and 100 μM), and the cultures were incubated for 24 h at 37°C. The well without any infection was used as the positive control and DMSO was used as the negative control.

### Statistical Analysis

The statistical analysis was accomplished by GraphPad Prism 6.0. Statistical differences with two test groups were analyzed using Student’s t-test. Asterisks indicated significance as follows:

* *p* < 0.05; ** *p* < 0.01; *** *p* < 0.001; **** *p* < 0.0001; ns, not significant.

## Results

### Evaluation of the Cytotoxicity of Cinchonine

**Figure 1:**
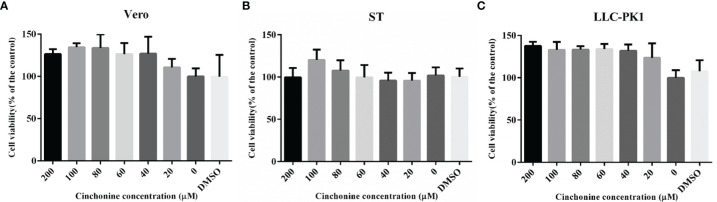
The cytotoxicity of cinchonine on Vero CCL81, ST and LLC-PK1 cells. **(A)** Vero CCL81 cell, **(B)** ST cell or **(C)** LLC-PK1 cell viability was evaluated by CCK-8 assay. All the values were normalized to the control, which represents 100% cell viability. The representative result of at least three independent experiments has been indicated. The data has been shown as mean ± standard deviation (SD).

To determine the potential antiviral activities of cinchonine against PEDV, we first used the CCK8 assay to evaluate the safe concentrations of cinchonine that can be used against Vero CCL81 cells, ST cells and LLC-PK1 cells. The cells were treated with different concentrations of the cinchonine ranging from 0 to 200 μM for 48 h, respectively. The results of CCK8 assay showed that no apparent cytotoxicity of cinchonine were observed on Vero CCL81 cells, ST cells and LLC-PK1 cells at a concentration of less than or equal to 200 μM (**Figures 1A-C**).

### Antiviral Effect of Cinchonine on PEDV Infection in Vero CCL81 and LLC-PK1 Cells

**Figure 2:**
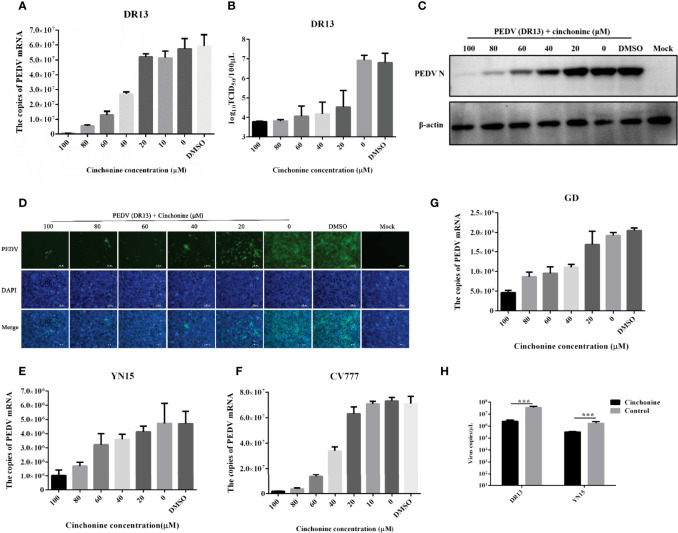
Detection of anti-PEDV activity of cinchonine in Vero CCL81 and LLC-PK1 cells. **(A–D)** Vero CCL81 cells were infected with DR13 strain in the presence of cinchonine at the various concentrations (0, 20, 40, 60, 80, 100 μM). At 24 h post-infection, the samples were prepared for the quantitative real-time RT-PCR, TCID_50_ assay, Western Blot or IFA. **(E–G)** Vero CCL81 cells were infected with CV777, YN15 or GD strain and then treated with the different concentrations (0, 20, 40, 60, 80, 100 μM) of cinchonine for 24 h. The mRNA level of PEDV was then measured by the quantitative real-time PCR. **(H)** LLC-PK1 cells were infected with DR13 or YN15 strain in the presence or absence of cinchonine. The samples were collected at 24 h to assess the copies of PEDV mRNA. The results depicted shown in Figures are representative result from at least three independent experiments. The data has been shown as mean ± standard deviation (SD). *** p < 0.001.

To explore the effect of different doses of cinchonine on the proliferation of the PEDV, Vero CCL81 cells were treated with cinchonine at a dose of 20, 40, 60, 80 or 100 μM or DMSO, and then infected with PEDV (MOI of 0.01), respectively. At 24 h post-infection, the cells were harvested and thereafter used for further experiments. To analyze the inhibitory effect of cinchonine on PEDV DR13 infection, the mRNA level of PEDV in cells and their supernatants were measured by quantitative real-time PCR. The results showed that cinchonine treatment significantly decreased PEDV mRNA levels in a dose-dependent manner (**Figure 2A**), thereby indicating that cinchonine can effectively inhibit PEDV replication. Meanwhile, the TCID_50_ results demonstrated that the titer of PEDV DR13 was significantly reduced as compared to the controls (**Figure 2B**). To further analyze the effect of cinchonine on PEDV protein synthesis, Western Blot assay was performed to detect the level of PEDV N protein in infected cells. As shown in **Figure 2C**, as the concentration of cinchonine increased, the expression level of N protein decreased gradually in infected cells, and moreover cinchonine at a dose of 80 μM or 100 μM nearly blocked the PEDV N protein synthesis completely. Finally, we applied IFA to determine the potential inhibitory effect of cinchonine on the number of PEDV infected cells. As a result, the PEDV specific immunofluorescence gradually reduced in infected Vero CCL81 cells (**Figure 2D**), thereby clearly suggesting that the antiviral effects of cinchonine exhibited a concentration-dependent response. To further verify that cinchonine displayed anti-PEDV effects, CV777, YN15 and GD were used to examine the antiviral effects of cinchonine, excluding the specificity of PEDV DR13 strain. As shown in **Figures 2E-G**, cinchonine could substantially reduce the mRNA level of PEDV infected cells in a dose-dependent manner. In addition, cinchonine had similar inhibitory effects against PEDV in LLC-PK1 cells (**Figure 2H**). Based on the above experimental results of cinchonine against PEDV, the anti-PEDV activity of cinchonine was confirmed.

### Evaluation the Effect of Cinchonine of the PEDV Life Cycle in Vero CCL81 Cells

**Figure 3:**
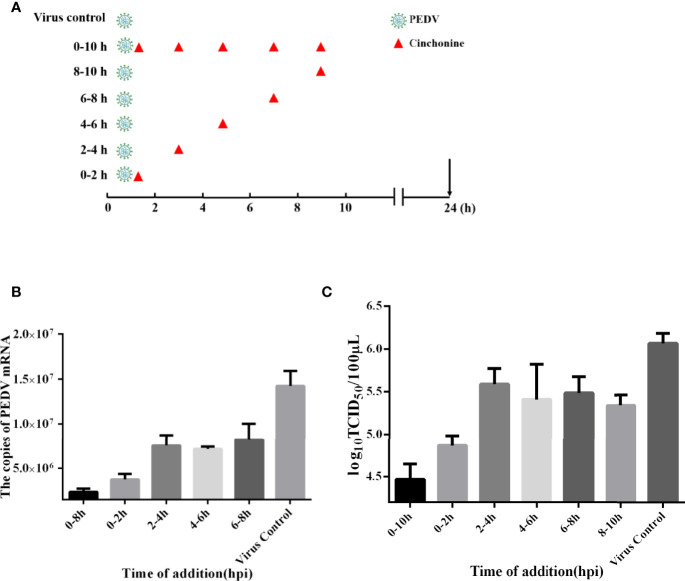
Time-of-addition effect of cinchonine on PEDV replication in Vero CCL81 cells. **(A–C)** Vero CCL81 cells were infected with the PEDV, and 100 μM cinchonine was added into infected cells at different time periods of PEDV infection: 0-10, 0-2, 2-4, 4-6, 6-8 and 8-10 h At 24 h, samples were prepared and examined with the quantitative real-time PCR and TCID_50_ assay. PC: virus control without cinchonine treatment. The data has been presented as the mean ± SD of at least three independent experiments.

The life cycle of PEDV includes adsorption, penetration, replication, assembly, and release (Fehr and Perlman, 2015). In order to in-depth explore the detailed mechanism of action of cinchonine in inhibiting PEDV infection, the time-of-addition assay was adopted to evaluate the potential stage of PEDV infection that was specifically affected by cinchonine. As shown in **Figure 3A**, vero CCL81 cells were exposed to PEDV and then treated with cinchonine (100 μM) for the indicated periods of time (0-10, 0-2, 2-4, 4-6, 6-8 and 8-10 h). At 24 h post-infection, the samples were collected for TCID_50_ assay and the quantitative real-time PCR. As shown in **Figures 3B, C**, the virus titers and mRNA levels were significantly reduced in the presence of cinchonine between 0-10 h. In contrast, cinchonine exhibited the strongest anti-PEDV activity when it was added at 2 h, thereby demonstrating that cinchonine may primarily affect PEDV adsorption and invasion to cells. In conclusion, these results indicated that cinchonine may exhibit a repressive inhibitory effect during the early stages of PEDV infection.

### Effect of Cinchonine on Autophagy in Vero CCL81, ST and LLC-PK1 Cells

**Figure 4:**
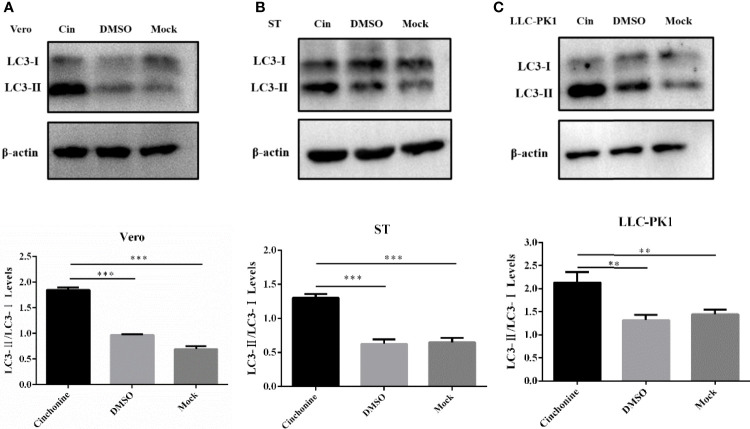
Expression of autophagy marker protein LC3 in cinchonine-treated cells. **(A–C)** Vero CCL81 cells, ST cells and LLC-PK1 cells were treated with 100 μM cinchonine for 24 h respectively, and the LC3 conversion was examined by Western Blot. The LC3-II/LC3-I levels were calculated by Image-Pro Plus 6.0 software. The above data has been expressed as the mean ± SD of at least three independent experiments. p < 0.05; ** p < 0.01; *** p < 0.001; **** p ≤ 0.0001; ns, not significant.

To elucidate the anti-PEDV activity of cinchonine *in vitro*, we further performed experiments to explore its underlying antiviral mechanism. We have convincingly demonstrated from the above findings that cinchonine could significantly attenuate the replication of PEDV on Vero CCL81 and LLC-PK1 cells. Existing studies have confirmed that rapamycin could also inhibit PEDV infection in porcine intestinal epithelial cells by inducing autophagy (Ko et al., 2017), so we hypothesized whether inhibitory actions of cinchonine on PEDV infection may also be related to autophagy. The autophagy protein LC3, which is involved in the formation of autophagosomal vacuoles, is widely used as an autophagosomal marker protein to monitor autophagy. When the autophagy occurs, LC3-I can combine with phosphatidyl ethanolamine (PE) to form LC3-II, which is located on the autophagosome double-layered membrane. Accordingly, autophagy can be monitored by measurement of the expression of LC3-II and conversion of LC3-I to LC3-II proteins (Rusmini et al., 2019). Vero CCL81 cells, ST cells and LLC-PK1 cells were treated with 100 μM cinchonine for 24 h respectively, and the protein expression of LC3 was examined by Western Blot. The results showed that there was a remarkable increase in LC3-II protein level and the conversion of LC3-I to LC3-II was also markedly increased as compared to the negative control group (**Figures 4A-C**). These findings indicated that cinchonine may regulate intracellular autophagy in Vero CCL81, ST and LLC-PK1 cells.

### Effect of Cinchonine on ER Stress in Vero CCL81 Cells, ST Cells and LLC-PK1 Cells

**Figure 5:**
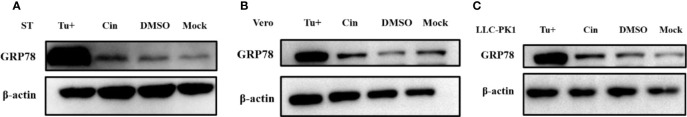
Expression of ER stress marker protein GRP78 in cinchonine-treated cells. **(A–C)** Vero CCL81 cells, ST cells and LLC-PK1 cells were treated with 100 μM cinchonine for 24 h respectively, and the expression of GRP78 protein was examined by Western Blot analysis.

A number of previous studies have indicated that ER stress inducers can display significant antiviral activities against PEDV (Wang et al., 2014). Interestingly, as cinchonine has been reported to activate ER stress through increasing the expression of GRP78/CHOP in human liver cancer cells especially in HeLa and A549 cells (Qi et al., 2017), we further identified whether cinchonine could stimulate ER stress in Vero CCL81 cells, ST cells and LLC-PK1 cells. Glucose regulating protein 78 (GRP78) is the primary protein that can regulate the refolding or degradation mechanism of misfolded proteins in ER (Elfiky et al., 2020). It is main player involved in the regulation of the unfold protein response (UPR) in ER and its upregulation can act as a potential marker of ER stress (Sun et al., 2021). Vero CCL81 cells, ST cells and LLC-PK1 cells were exposed to cinchonine for 24 h, and the expression of GRP78 protein was detected by Western Blot analysis. It was found that compared with the positive control (Tu, which is a mixture of antibiotic that can inhibit N-linked glycosylation which causes accumulation of unfold proteins and activates ER stress, cinchonine treatment group did not lead to an increase the expression of GRP78 protein effectively, thus demonstrating that cinchonine could not induce ER stress in Vero CCL81 cells, ST cells and LLC-PK1 cells (**Figures 5A-C**). The above results suggested that cinchonine might activate ER stress only in a cell-specific manner and is not a universal ER stress activator.

### Cinchonine Inhibits PEDV Infection in Vero CCL81 Cells Through Autophagy

**Figure 6:**
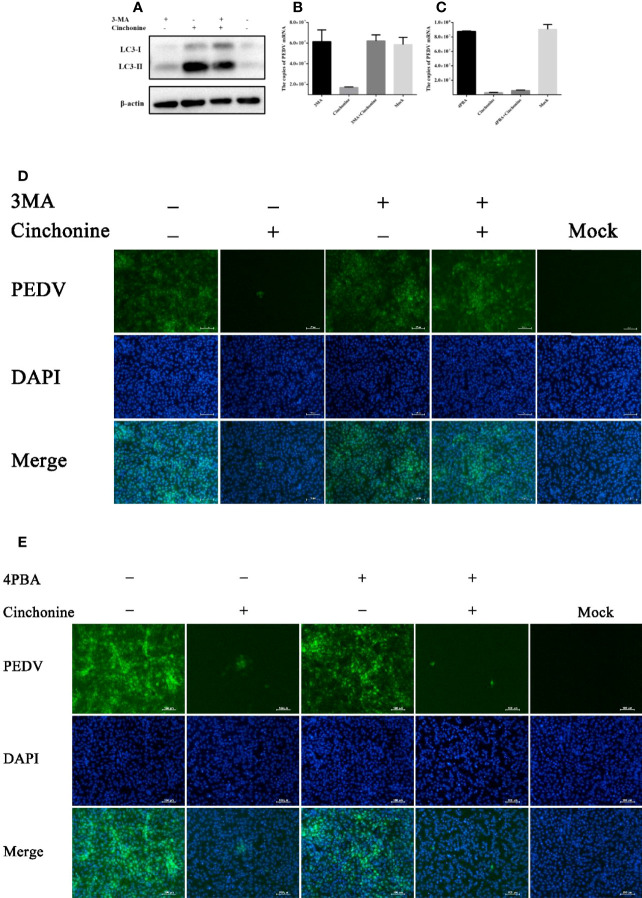
Cinchonine inhibits PEDV infection in Vero CCL81 cells through inducing autophagy. **(A)** Vero CCL81 cells were treated with or without 3 mM 3MA for 2 h prior to 100 μM cinchonine treatment for 24 h, and then the expression of LC3 was analyzed by Western Blot analysis. **(B–E)** Vero CCL81 cells incubated with or without of 3MA (3 mM) or 4-PBA (100 μg/mL) were treated with 100 μM cinchonine, and then infected with PEDV. The samples were collected at 24 h post infection to estimate the mRNA level of PEDV by the quantitative real-time PCR, and the number of PEDV infected cells was thereafter evaluated by IFA. The data has been represented as the mean ± SD of at least three independent experiments.

Since cinchonine could induce autophagy, we further analyzed the potential effect of cinchonine on PEDV infection in the presence of 3MA. 3MA is a PI3K inhibitor, which is widely used to inhibit autophagy *via* its inhibitory effect on class III PI3K (Miller et al., 2010). Vero CCL81 cells were pretreated with 3MA for 2 h and then infected with PEDV in the presence or absence of 3MA for 24 h. As shown in **Figure 6A**, the expression of LC3-II protein was markedly reduced in 3MA treatment group as compared with the 3MA untreated group. In addition, when autophagy was inhibited, we observed that the numbers of PEDV infected cells (**Figure 6D**) and mRNA levels of PEDV (**Figure 6B**) were significantly attenuated in the presence of cinchonine and that the infection of PEDV was rescued in the presence of 3MA. These results revealed that autophagy inhibitor 3MA could significantly alleviate the reduction of PEDV infection caused by cinchonine.

It has been reported that cinchonine could activate ER stress, so we next analyzed the effect of cinchonine on PEDV replication in the presence of ER stress inhibitor. We pretreated Vero CCL81 cells with 4-Phenylbutyric acid (4-PBA), which has been reported to relieve ER stress by inhibiting the phosphorylation of eIF2α (Ozcan et al., 2006; Ozcan et al., 2008). As shown in **Figures 6C, E**, no significant changes in the numbers of PEDV infected cells and the mRNA levels of PEDV were observed compared with the group treated with cinchonine without 4-PBA, thereby indicating that 4-PBA was not able to effectively mitigate PEDV infection inhibition by cinchonine.

Collectively, these findings supported our hypothesis that autophagy induced by cinchonine was primarily responsible for the inhibition of PEDV infection.

## Discussion

The outbreak of porcine epidemic diarrhea can result in a high fatality rate in newborn piglets, which has resulted in a huge economic burden to the global swine industry. Although vaccines can be used to prevent PEDV, commercial vaccines fail to provide protection against highly pathogenic PEDV variants (Li et al., 2016; Chen et al., 2017). Hence, novel therapeutic drugs are urgently needed to control PEDV in the clinic, and effective antiviral drugs can play an important role in preventive treatment. Several agents that can adversely affect PEDV infection have been reported previously. For example, quercetin, a flavonoid molecule, has been reported to suppress PEDV replication in Vero CCL-81 cells by inhibiting PEDV 3C-Like protease activity (Li et al., 2020). In addition, Glycyrrhizin (GLY) extracts from licorice can restrain PEDV infection by relying on the HMGB1/TLR4-MAPK p38 pathway to prevent binding of HMGB1 to TLR4 (Gao et al., 2020). However, the antiviral mechanisms identified in these studies were all different. Here we report that cinchonine-induced autophagy can negatively regulate the proliferation of PEDV *in vitro*.

Cinchonine is considered to be one of the most valuable alkaloids in cinchona bark. Cinchona bark has been found to have remarkable biological properties at the beginning of 20^th^ century, however, it was not commercialized due to the significant changes in the composition of the bark’s crude extract (Rankovic et al., 2019). For instance, quinine can display potent antiviral activity against dengue virus (DENV) by blocking viral RNA and protein synthesis, and it can also contribute to the upregulation of the various antiviral genes (Malakar et al., 2018). Intriguingly, quinine can restrain infection of human cells lines with SARS-CoV-2, especially in TMPRSS2+ human lung cancer cell lines (Große et al., 2021). Apart from quinine, the three other alkaloids, cinchonine, quinidine, and cinchonidine, have not been investigated for their potential antiviral activities. Some studies have reported that cinchonine possesses antimalarial (Sowunmi et al., 1990) and antitumor potential, but its efficacy against viral diseases has not been analyzed. Therefore, we aimed to elucidate the antiviral properties of cinchonine in this study.

In the present study, we firstly found that cinchonine displayed prominent antiviral activity against different strains of PEDV, and its antiviral effects were primarily observed in the early stages of the viral life cycle, which encouraged us to further explore its anti-PEDV mechanism. In the last few years, the relationship between autophagy and coronavirus infections has been extensively studied, and coronavirus not only can induce but inhibit autophagy (Singh et al., 2021). It has been reported that modulation of autophagy inhibits coronavirus infection, making it a promising target for anticoronavirus treatment. For example, autophagy inhibitor chloroquine blocked autophagic flux and prevented the formation of autophagosome, thereby inhibiting the replication of SARS-CoV and SARS-CoV-2 (Vincent et al., 2005; Mauthe et al., 2018; Yao et al., 2020). In addition, autophagy induced by rapamycin can suppress the infection of MERS-CoV and TGEV (Kindrachuk et al., 2015; Guo et al., 2016). In this study, we found that autophagy induced by cinchonine can suppress the replication of PEDV in Vero and LLC-PK1 cells. These results were consistent with previous study on rapamycin-induced autophagy in IPEC-J2 cells ultimately inhibited PEDV proliferation by Ko S. et al. (Ko et al., 2017). Moreover, which proteins (structural or nonstructural proteins) of PEDV are the critical inducers of autophagy has been extensively studied. Lin H. *et al.* reported that nsp6 was one of the pivotal inducers of PEDV-mediated autophagy, which mainly worked *via* PI3K/Akt/mTOR signaling pathway (Lin et al., 2020). PEDV ORF3-induced autophagy was dependent on ER stress response through up-regulation of GRP78 and activation of PERK-eIF2α signaling pathway (Zou et al., 2019). Thus, the role of autophagy in PEDV replication may differ in different cells, and the outcome of autophagy and viral replication may be intricately regulated by multiple signaling pathways. It has also been investigated that beclin 1 is closely related to the degradation process after the fusion of autophagosome and lysosome (Glick et al., 2010). The membrane-associated PLP2 of PEDV induces an incomplete autophagy, suggesting that it may impairs the degradation of autolysosome or disturb the fusion of autophagosome and lysosome (Chen et al., 2014; Ko et al., 2017). Therefore, understanding whether cinchonine can act as an inhibitor of the papain-like protease (PLP) domain of PEDV to inhibit its activity, or whether cinchonine can upregulate beclin1 protein expression to promote the formation of autophagolysosome require to in depth research.

Many enveloped RNA viruses could infect mammalian cells and induce ER stress. For instance, studies on TGEV have shown that TGEV infection can induce ER stress *in vitro* and *vivo*, which could potentially activate the PERK-eIF2α branch to regulate TGEV replication negatively. However, some viruses can exploit ER stress to enhance their replication. PRRSV infection can induce ER stress and UPR in Marc-145 cells by activating PERK and IRE1 pathways, but not affecting ATF6 pathway. UPR induction by PRRSV might facilitate their replication. In the studies on PEDV, it was demonstrated that 2-Deoxy-D-glucose (2-DG) can trigger UPR which in turn can lead to the suppression of PEDV infection *in vitro*. In addition, further studies have indicated that cinchonine could activate ER stress through promoting PERK and eIF2α phosphorylation (Jin et al., 2018). This aroused our interest to explore whether cinchonine also can exhibit its anti-PEDV by activating ER stress. Surprisingly, we discovered that the antiviral activity of cinchonine might be associated with induction of autophagy rather than ER stress, as shown in our reported results.

In conclusion, this study represents the first report describing that cinchonine can induce autophagy to inhibit PEDV infection. Our study has highlighted the outstanding antiviral activities of cinchonine against PEDV *in vitro*, thus suggesting that activation of autophagy might serve as an important protective strategy and provide new strategies for the development of novel antiviral agents.

## Data Availability Statement

The original contributions presented in the study are included in the article/supplementary material. Further inquiries can be directed to the corresponding author.

## Author Contributions

Conceptualization and writing: JR and QH; Data curation: WZ and JR; Funding acquisition: QH; Investigation: JR, WZ, CJ, CL, CZ, HC, WL, and QH; Methodology: JR, WZ, and QH; Supervisor: QH; Writing-original draft: JR. All authors contributed to the article and approved the submitted version.

## Funding

This project was particularly grateful for the support of the National Natural Science Foundation of China (31972667).

## Conflict of Interest

The authors declare that the research was conducted in the absence of any commercial or financial relationships that could be construed as a potential conflict of interest.

## Publisher’s Note

All claims expressed in this article are solely those of the authors and do not necessarily represent those of their affiliated organizations, or those of the publisher, the editors and the reviewers. Any product that may be evaluated in this article, or claim that may be made by its manufacturer, is not guaranteed or endorsed by the publisher.
